# Prefrontal cortex hemodynamics and age: a pilot study using functional near infrared spectroscopy in children

**DOI:** 10.3389/fnins.2014.00393

**Published:** 2014-12-15

**Authors:** Afrouz A. Anderson, Elizabeth Smith, Victor Chernomordik, Yasaman Ardeshirpour, Fatima Chowdhry, Audrey Thurm, David Black, Dennis Matthews, Owen Rennert, Amir H. Gandjbakhche

**Affiliations:** ^1^National Institutes of Health, Eunice Kennedy Shriver National Institute of Child Health and Human DevelopmentBethesda, MD, USA; ^2^Department of Biomedical Engineering, University of California, DavisDavis, CA, USA; ^3^National Institute of Mental HealthBethesda, MD, USA; ^4^Department of Neurological Surgery, School of Medicine, University of California, DavisDavis, CA, USA

**Keywords:** functional near infrared spectroscopy, autoregulation, hemodynamic model

## Abstract

Cerebral hemodynamics reflect cognitive processes and underlying physiological processes, both of which are captured by functional near infrared spectroscopy (fNIRS). Here, we introduce a novel parameter of Oxygenation Variability directly obtained from fNIRS data —the OV Index—and we demonstrate its use in children. fNIRS data were collected from 17 children (ages 4–8 years), while they performed a standard Go/No-Go task. Data were analyzed using two frequency bands—the first attributed to cerebral autoregulation (CA) (<0.1 Hz) and the second to respiration (0.2–0.3 Hz). Results indicate differences in variability of oscillations of oxygen saturation (SO_2_) between the two different bands. These pilot data reveal a dynamic relationship between chronological age and OV index in CA associated frequency of <0.1 Hz. Specifically, OV index increased with age between 4 and 6 years. In addition, there was much higher variability in frequencies associated with CA than for respiration across subjects. These findings provide preliminary evidence for the utility of the OV index and are the first to describe the relationship between cerebral autoregulation and age in children using fNIRS methodology.

## Introduction

Due to its high metabolic demand and dynamic nature, the brain requires a rapid yet precise mechanism of oxygen delivery. Since oxygen is transported by means of binding to hemoglobin molecules, local concentrations of hemoglobin species (i.e., both oxy- and deoxy-hemoglobin) represent the oxygen-carrying capacity of corresponding regions of cerebral tissue. Temporal variations in the concentration of these species can therefore reveal the cerebral hemodynamics of functional brain activity. In particular, these variations provide information about cerebral autoregulation (CA), the mechanism that optimizes oxygen delivery to the brain. CA keeps cerebral blood flow (CBF) constant regardless of changes in atrial blood pressure, and is a vital mechanism for brain function (Clarke and Sokoloff, 1999; Van Beek et al., 2008; Cipolla, 2009).

Local changes in cerebral hemodynamic levels in the cortex can be evaluated non-invasively by functional near infrared spectroscopy (fNIRS) (Yodh and Boas, 2003; Boas et al., 2004; Gratton et al., 2005; Huppert et al., 2009; Benavides-Varela et al., 2011; Amyot et al., 2012). The NIR absorption spectrum of brain tissue is sensitive to changes in the concentration of major tissue chromophores, including hemoglobin species. Therefore, measurements of temporal variations of backscattered NIR light can capture functionally-evoked changes in the outermost cortex, and thus, can be used to assess hemodynamic variation. Compared to other well-established brain imaging modalities, such as fMRI and positron emission tomography (PET), fNIRS offers unique features, including higher temporal resolution (i.e., several milliseconds), and spectroscopic information about temporal variations for both components of hemoglobin, (i.e., Oxy-[HbO] and deoxy-[Hb]). fMRI BOLD signal is most sensitive to the magnetic property of deoxy-hemoglobin [Hb]. Importantly for pediatric applications, fNIRS instruments are small and transportable, less restraining compared to fMRI or PET, and can tolerate a large degree of subject motion (Riley et al., 2012). These features make the technique well- suited for young children who are not able to remain still for long periods of time (Elwell et al., 2005; Nagamitsu et al., 2006; Gervain et al., 2011).

Different physiological processes result in hemodynamic oscillations at specific characteristic frequencies (Sassaroli et al., 2012). For example, low frequency oscillations (LFO) at 0.07–0.1 Hz are known to be associated to CA in adults (Obrig et al., 2000; Van Beek et al., 2008; Sassaroli et al., 2012), while higher frequency oscillations at 0.2–0.3 Hz are known to be related to respiration (i.e., they result from corresponding vascular variations and blood pooling). However, the frequency range associated with autoregulation in children and infants likely ranges from 0 to 0.1 Hz (Bassan et al., 2005; Wong et al., 2008). Moreover, this broader frequency range also captures the influence of cerebral vasomotion and oscillation of CBF (Zhang et al., 1998; Müller and Osterreich, 2014). Finally, the effect of end-tidal CO_2_ (EtCO_2_) at lower frequency bands (<0.07) has been demonstrated during hypercapnia, although during normocapnia the changes in cerebral autoregulation may not be influenced by EtCO_2_ (Panerai et al., 2005; Liu et al., 2010; Maggio et al., 2014). Spontaneous oscillations, induced by CA in tone of blood vessels (due to vasomotion), are not related to heart pulsation (1–1.5 Hz) or respiration (0.2–0.3 Hz) (Naseer and Hong, 2013; Pierro et al., 2014), and they can potentially become a predictor of degree of autoregulation and its impairment.

CA has been a focus of several recent studies, due to its importance in brain function and metabolism, and its ability to predict both normal and abnormal autoregulation (Bassan et al., 2005; Wong et al., 2008). For example, Tsuji et al. (2000) and Caicedo et al. (2011) used NIRS to monitor cerebral circulation and blood flow in infants with impaired CA. These studies found high correlations between cerebral intravascular oxygenation and mean atrial blood pressure (MAP), a marker of autoregulation impairments.

Age-related changes in cerebral hemodynamics have been reported in other modalities that measure cerebral blood flow (CBF, i.e., Chiron et al., 1992; Schöning and Hartig, 1996; Takahashi et al., 1999; Biagi et al., 2007; Kilroy et al., 2011). For instance, Schöning et al. used color duplex sonography of the internal carotid and vertebral arteries to investigate cerebral hemodynamics. This study found an increase in CBF from age 3 up until 6.5, and a subsequent decline that reached a constant level at approximately 15 years. Other studies using arterial spin labeling (ASL) have also found that CBF decreases with age in children over 7. Moreover, the decrease in CBF appears to continue throughout adolescence and adulthood (Biagi et al., 2007; Kilroy et al., 2011). PET measurements in children also reveal an increase in regional CBF with children's age peaking at the age of 7 and a subsequent decline in older children (Takahashi et al., 1999), with similar results using [^133^Xe]- SPECT technology (Chiron et al., 1992). While PET studies use injections of radioactive contrast to measure changes in blood flow, the present study of children uses the more suitable and feasible non-invasive fNIRS method to measure changes in hemodynamics at frequencies related to cerebral autoregulation.

In the current study, we use frequency analysis of oscillatory patterns from fNIRS to formulate a new metric of variations in local oxygen saturation. To characterize this metric, we introduce an Oxygen Variability Index (OV Index) and analyze two frequency bands (<0.1 Hz and 0.2–0.3 Hz). We then examine the relationship between OV Index in these two bands and age in a group of typically developing children. We hypothesize that the OV index, measured with the non-invasive and patient-friendly fNIRS modality, will reveal differences based on chronological age as seen in the extant literature on cerebral hemodynamics in children.

## Materials and methods

### Participants

Seventeen typically developing children between the ages of 4 and 8 (Female = 8, mean age = 5.99 ± 1.04) were recruited from the community for this IRB approved study. All children had non-verbal and verbal IQ scores of 80 or above. Exclusion criteria included contraindications for wearing the NIRS equipment, diagnosed psychiatric disorder, specific current medications for neuropsychiatric disorders, history of vascular disease, conditions affecting cerebral anatomy, head trauma, neurological or neurogenetic disorders, or premature birth. Subjects' parents/guardians consented to their child's participation. Subjects were first screened for inclusion and exclusion criteria by parent interview, parent report, and cognitive testing, (i.e., either the Differential Abilities Scale, 2nd edition (DAS, Elliott, 2007) or the Wechsler Preschool and Primary Scale of Intelligence, 4th edition (Wechsler, 2012), depending on subject age).

### Task

**Figure 1:**
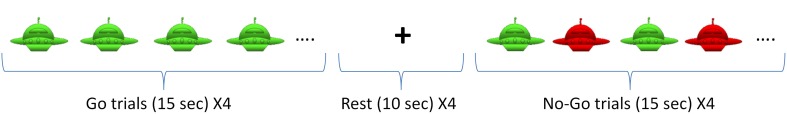
**Go/No-Go Task**. The task included 4 Go blocks and 4 No-Go blocks with alternating presentation as shown above.

Children completed the Go/No-Go task, a well-known response inhibition task that elicits functional activity in pre-frontal cortex (Simmonds et al., 2008). This task is simple enough for young children and also required significant attention. Subjects were asked to press a button only in response to the Go stimuli (green spaceships) and refrain from pressing when the No-Go stimuli (red spaceships) were presented. Alternating blocks of Go and No-Go (total of eight blocks) were used (see Figure 1). During the Go blocks, a series of green spaceships were shown, whereas during the No-Go blocks both red and green spaceships were presented randomly with a green to red ratio of 10:4. Stimulus duration was 500 ms with 1000 ms of interstimulus interval. Each block was followed by resting period of 10 s. The task related frequency was chosen at 0.66 Hz to prevent the overlap with frequency bands of interest (<0.1 Hz and 0.2–0.3 Hz).

### Data acquisition and analysis

**Figure 2:**
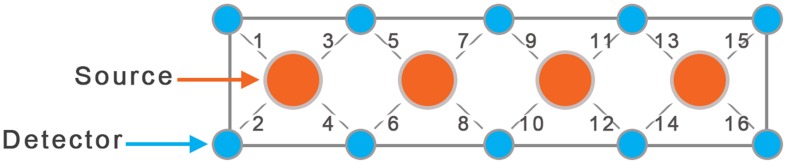
**Schematic of fNRIS sensor, using 4 sources and 10 detectors**.

In this study we used a continuous wave fNIRS system (fNIR Devices LLC, Potomac, MD). The instrument consists of an array of 4 sources and 10 detectors, with a total of 16 source-detector pairs (see Figure 2). It collects data at two wavelengths—730 and 850 nm—with an acquisition frequency of 2 Hz. The device is equipped with low pass filter for the analog signal to prevent aliasing problems, where higher frequencies can be aliased into the lower band. Light is delivered at the source position, and backscattered light is measured at the position of the detector, with a fixed source-detector distance of 2.5 cm. Therefore, the same source-detector distance was used across all subjects. Using two wavelengths allows one to probe regional cortical changes in both Oxy- and Deoxy-hemoglobin concentrations.

The sensor was positioned on each child's forehead covering the prefrontal cortex (PFC) area of BA 9 and 10, and the amount of activation was characterized by changes in concentration of oxy- and deoxy-hemoglobin. Due to smaller head size in children only the 8 middle channels (5–12) were used for data collection.

The NIR light intensities at two wavelengths were then converted to changes in oxy- and deoxy-hemoglobin concentration using fNIRSOFT software (Ayaz, 2010). Here, the modified Beer-Lambert law (Cope, 1991) was used for calculating the changes in oxy- and deoxy-hemoglobin using the 10 s of baseline measurement before the task's onset.

To analyze cerebral hemodynamics at specific frequency bands, we applied IIR frequency filters (butterworth, order 10) to the whole raw continuous temporal Oxy- and Deoxy-hemoglobin signals. Then, using the filtered data, blocks of Go, No-Go, and rest were separately averaged over the duration of task. Blocks were visually inspected and those containing motion artifacts, characterized by high amplitude spikes, were excluded from averaging. Such exclusion happened for three subjects, with a total of one block per subject. Instantaneous amplitude *A*(*t*) and phase φ(*t*) of Oxy- and Deoxy-hemoglobin variations were obtained from the filtered NIRS data (in a given narrow frequency band) using an algorithm, based on an analytic signal continuation approach that can be described by the following formula (Boashash, 1992):

$$v\left(t\right)=S\left(t\right)+jH\left|S\left(t\right)\right|=A\left(t\right)e^{-j\varphi \left(t\right)}$$

where *S*(*t*) is the real signal, *H* denotes the Hilbert transform and *v*(*t*)indicates complex signal in the time domain.

Combining the data for both hemoglobin species we were able to quantify instantaneous oxygen saturation $SO_{2}(t):SO_{2}=\frac{\left[Hbo\right]}{\left[Hbo\right]+\left[Hb\right]}$, where [*HbO*] and [*Hb*] are amplitudes of analytic signal in the given frequency band, corresponding to both hemoglobin species. Hence, the value of oxygen saturation is based on ratio of changes in oxy- and deoxy-hemoglobin. We applied this technique to both <0.1 Hz and respiration induced oscillations in the frequency range of 0.2–0.3 Hz.

To quantify oscillations in oxygen saturation, we used the oxygenation variability index (*OV* index) as the dimensionless ratio $OV=\frac{\sigma \left(SO_{2}\right)}{\mu \left(SO_{2}\right)}$, where σ(SO_2_) and μ(SO_2_) are the standard deviation and mean values of the evaluated oxygen saturation signal (SO_2_), respectively. In other words, the *OV* index characterizes the level of variability of the oxygen saturation (SO_2_) for both <0.1 Hz and 0.2–0.3 Hz frequency bands.

Statistical analyses included paired *t*-tests for behavioral data, regression analyses to determine the relationship between the OV and age, and repeated measures ANOVA to analyze the effect of condition on OV index.

## Results

### Behavioral data

All trials with reaction times between 100 and 1000 ms were included in analyses. Table 1 displays reaction times (RT) for both the Go and No-Go blocks along with means and standard deviations for true hit percentage, false alarm percentage, and overall accuracy. Subjects' reaction times were significantly slower during No-Go than Go blocks [statistics performed with 1/x transformed values due to expected positive skew of RT data, *t*_(16)_ = 5.7, *p* < 0.001]. False alarm percentage was higher than zero [*t*_(16)_ = 8.1, *p* < 0.001], and lower than correct Go hits for the No-Go blocks [*t*_(16)_ = −13.8, *p* < 0.001].

**Table 1:** **Descriptive Statistics for Go/No-Go task for all subjects**.

**Measure**	**Mean (*SD*) [range]**	**Measure**	**Mean (*SD*) [range]**
Go RT (ms)	448 (83) [338–560]	No-Go Green hit (%)	91 (11) [65–100]
No-Go RT (ms)	487 (86) [378–637]	No-Go False Alarm (%)	26 (13) [6–62]
		No-Go Accuracy (%)	84 (9.6) [61–95]

*RT, reaction time. Accuracy is calculated as number of correct green hits plus correct red misses divided by total number of trials. False alarm percent is total red hits divided by the number of red trials. Green hit percent is the number of green hits divided by the number of green trials presented*.

### OV index and age

**Figure 3:**
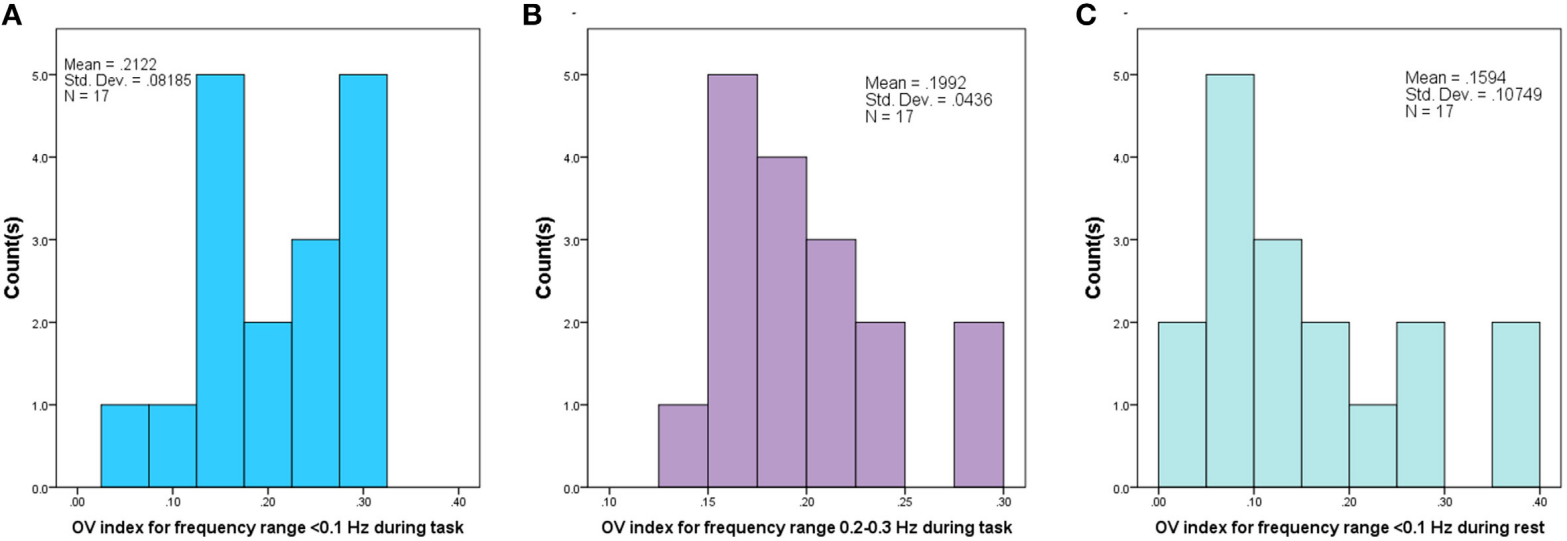
**Histogram of Oxygenation Variability Index (OV index) based on Coefficient of Variation for 17 subjects, corresponding to (A) Frequency range (<0.1 Hz) during performance of the task, (B) Frequency range (0.2–0.3 Hz) corresponding to respiration during the task and (C) for Frequency range (<0.1 Hz) during rest period**.

Data across all conditions were examined for normality and homogeneity of variance. The Shapiro-Wilk test for non-normality revealed distributions of the OV index, for frequencies <0.1 Hz, during the task and for the respiration frequency did not violate assumptions of normality (*p* = 0.45 and *p* = 0.31, respectively) (see Figure 3). The test also revealed a non-normal distribution for the OV index for frequencies <0.1 Hz during rest (*p* = 0.041). Further, variance did not differ among the conditions (Go, No-Go, and Rest) for the OV Index, for frequencies <0.1 Hz, (Levene Statistic = 0.27, *p* = 0.76). ANOVA is robust to departures from normality when the assumption of homogeneity of variance is not violated, so parametric tests were used when investigating these conditions.

We first investigated the relationship between age and OV Index, for frequencies <0.1 Hz, across the Go, No-Go, and rest conditions, and did not find a significant linear effect for age [*F*_(15)_ = 0.19, *p* = 0.67]. We then examined the relationship between age and OV Index for nonlinear trends, and found a significant quadratic relationship, for frequencies <0.1 Hz, across all conditions [*F*_(14)_ = 10.6, *p* = 0.006]. We investigated both linear and nonlinear trends for the respiration frequencies, and found no significant effect of age [Linear: *F*_(15)_ = 0.98, *p* = 0.34; Quadratic: *F*_(14)_ = 0.01, *p* = 0.91].

In order to determine the relative effects of the quadratic effect of age and condition, we then used a repeated measures ANOVA with age as the between subjects-factor and condition (Go, No-Go, and Rest) as the within-subjects factor. There was no condition by age interaction [*F*_(14)_ = 0.73, *p* = 0.5]. Thus, we removed the non-significant interaction from the model and found a significant main effect for condition after accounting for the quadratic effect of age [*F*_(16)_ = 5.0, *p* = 0.02]. *Post-hoc* comparisons showed higher OV Index, for frequencies <0.1 Hz, for both No-Go and Go conditions when compared to rest [*F*_(16)_ = 5.3, *p* = 0.04, *F*_(16)_ = 8.3, *p* = 0.01, respectively], but no difference in OV Index between No-Go and Go conditions [*F*_(16)_ = 0.25, *p* = 0.62].

**Figure 4:**
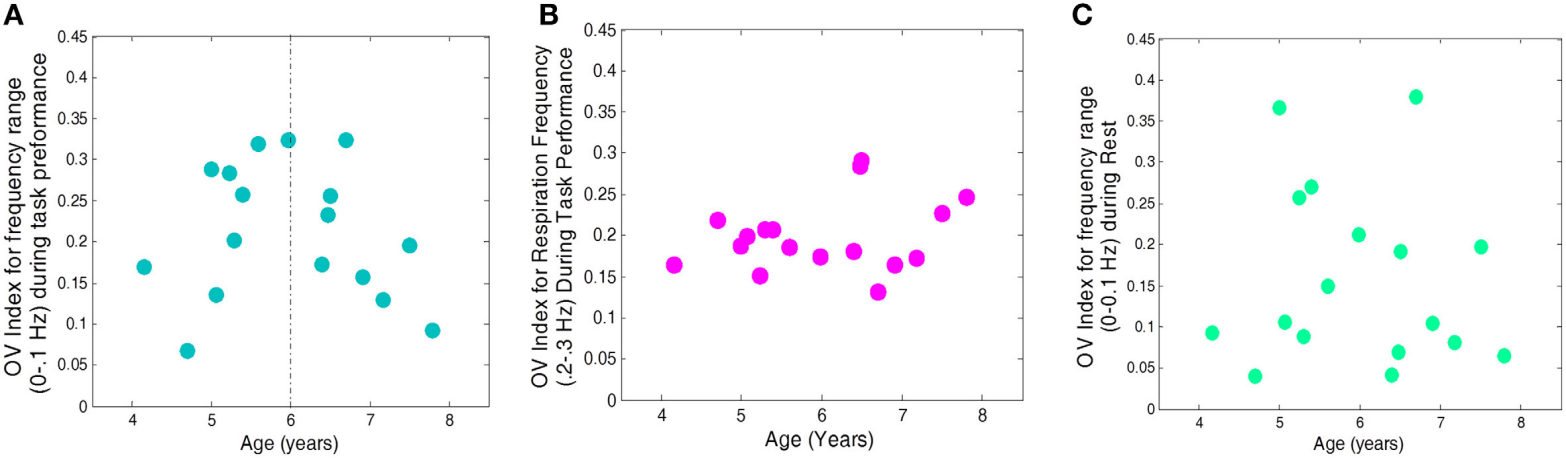
**OV index vs. Age for 17 subjects. (A)** for frequencies related to autoregulation (<0.1 Hz) during the task (collapsed across No-Go and Go conditions), **(B)** for frequencies related to respiration (0.2–0.3 Hz) during the task, and **(C)** for frequencies related to autoregulation (<0.1 Hz) during rest.

**Figure 5:**
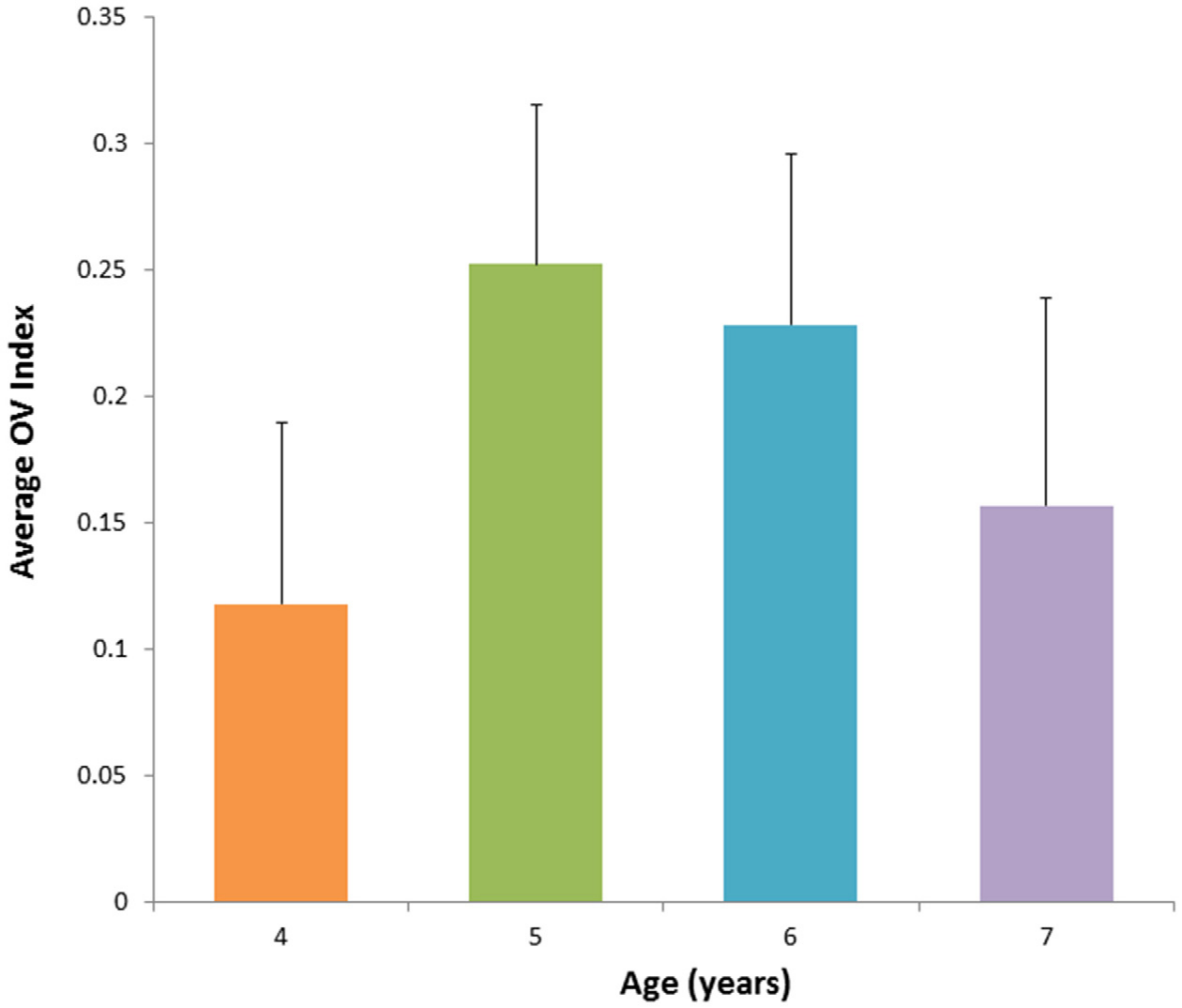
**Average value for OV Index across age groups corresponding to the frequency band <0.1 Hz during performance of the task**. The OV index increases with age between 4 and 6 years and is followed by subsequent decrease with age.

After establishing a statistically similar OV index values for the Go and No-Go conditions, we combined the two into one condition, “Task,” and reanalyzed the relationship with age. Across all conditions (task, rest), the quadratic effect of age remained significant [*F*_(14)_ = 9.94, *p* = 0.007]. We also investigated whether representing data in this way would reveal a different relationship between condition, age, and OV index, but found no significant interaction between condition (task vs. rest), age, and OV index [*F*_(14)_ = 1.16, *p* = 0.30]. The above quadratic fit reveals a maximum at 5.94 years; therefore, we examined linear trends for children above and below 6 years. OV index increased significantly with age for children between the ages of 4 and 6 years (*r* = 0.68, *p* = 0.039) and showed a trend toward decreasing with age for children ages 6–8 years (*r* = −0.62, *p* = 0.1, see Figures 4, 5). The OV index did not show a significant correlation with age for either age group during rest (below 6, *r* = 0.34, *p* = 0.35; ages 6–8, *r* = −0.09, *p* = 0.81).

## Discussion

Functional brain studies can potentially serve as a guide for understanding the dynamics of the brain function and cerebral autoregulation. We focused our analysis on two frequency bands, <0.1 Hz and 0.2–0.3 Hz, which are related to cerebral autoregulation and respiration, respectively. To quantify the observed hemodynamic oscillations in the above frequency bands, we have introduced the Oxygenation Variability Index (OV index) as a metric to assess degree of CA in this sample of 4–8 year old children. In this study, we saw more variation in the OV index than in the respiration frequency. A tighter distribution in the respiration frequency band likely indicates similar breathing patterns across subjects.

Variations in OV index in children indicated a dynamic relationship between age and hemodynamic CA oscillations in frequency band below 0.1 Hz. Specifically, there was a significant quadratic effect of age that included a parabola peaking just before 6 years of age (5.94 years). The linear fit for the group of children below age 6 also indicated a strong linear correlation between age and OV index for ages 4–6. The significant relationship between age and OV index is in line with studies using other modalities showing a similar relationship between CBF and age. Specifically, those studies show CBF increasing until about 6–7 years and decreasing afterward and eventually reaching a plateau. (Chiron et al., 1992; Schöning and Hartig, 1996; Takahashi et al., 1999; Biagi et al., 2007; Kilroy et al., 2011). Here, we characterize that pattern further by showing a quadratic trajectory of change in OV index in frequencies related to CA. Additional data, particularly longitudinal data, is needed to improve the power to model this effect. Expanding the sample to children over age 6 will also be important, in order to characterize this trajectory across development. Finally, future studies should include simultaneous measurement of other physiological processes related to CA, including breathing, heart rate, and ETCO_2_. This is necessary in order to investigate the role these processes play in CA, including variations in CA with increasing mental load.

The physiological reasons for an increase in CA peaking at age 6 are not fully understood. Peak values of the OV index at around age 6 years may be related to other neurophysiological changes occurring at that time requiring increased regulation of CBF. Future studies should examine the relationship between cerebral autoregulation and other measures of brain anatomy during development (e.g., structural changes in gray matter and white matter). It will also be important to measure CA in children younger and older than those in the current sample in order to further clarify the relationship between age and the OV index.

The task and rest were used in conjugation to see whether CA would show any differences related to performance of functional task conditions. Though the OV index, for frequencies <0.1 Hz, was similar during Go and No-Go conditions, OV Index was lower during rest. This finding is in line with those showing increases in efficiency of CA during completion of cognitive tasks (Panerai et al., 2005). While the current study focuses on the prefrontal cortex, future studies should include other functional tasks known to elicit activity in other brain regions in order to further investigate the link between OV index, age, and task. Such analyses might reveal a significant role for frequencies <0.1 Hz across a broad range of tasks/stimulus types and brain regions.

Overall, results of this pilot study indicate that the variability in OV Index is related to developmental changes of CA in children. Increasing the number of subjects and extending to older children, as well as additional measurements, such as heart rate, breathing and ETCO_2_ are necessary to improve validation of the results. In future studies, this index may ultimately be used to elucidate the relationship between CA development and brain function. Further, we show that the choice of frequency plays a crucial role in understanding physiological processes at different stages of physiological development. While analysis of hemodynamic oscillations originating from cerebral autoregulation does not provide a full picture of this complicated phenomenon, the non-invasive and patient-friendly fNIRS method can be used to monitor brain development and possibly detect impairments of cerebral autoregulation.

### Conflict of interest statement

The authors declare that the research was conducted in the absence of any commercial or financial relationships that could be construed as a potential conflict of interest.
